# Prognostic value of C-reactive protein to albumin ratio for mortality in acute kidney injury

**DOI:** 10.1186/s12882-023-03090-9

**Published:** 2023-02-25

**Authors:** Baohua Liu, Dezhao Lv

**Affiliations:** Department of Rehabilitation, the Second Affiliated Hospital and Yuying Children’s Hospital of Wenzhou Medical University, Wenzhou, 325000 Zhejiang China

**Keywords:** C-reactive protein, Albumin, Acute kidney injury, Mortality

## Abstract

**Background:**

Inflammation plays an important role in the development of acute kidney injury (AKI). However, there are few studies exploring the prognostic influence of C-reactive protein to albumin ratio (CAR) among AKI patients. In this study, we investigated whether CAR could be a useful marker to predict the mortality of AKI.

**Methods:**

A total of 358 AKI patients were extracted from the Medical Information Mart for Intensive Care III (MIMIC III) database. C-reactive protein (CRP) and albumin were measured at ICU admission. The clinical outcome was 365-day mortality. Cox proportional hazards model and Kaplan-Meier survival analysis were conducted to evaluate the association between CAR and outcome.

**Results:**

Compared with patients in the survival group, nonsurvivors had higher CAR levels. The area under the receiver operating characteristic (ROC) curve of CAR was higher than that of CRP and albumin for mortality (0.64 vs. 0.63, 0.59, respectively). The cut-off point of CAR for mortality was 7.23. In Cox proportional-hazard regression analysis, CAR (hazards ratio (HR) =2.04, 95% confidence interval (CI) =1.47-2.85, *p* < 0.001 for higher CAR) and Simplified Acute Physiology Score II (HR = 1.02, 95%CI = 1.00-1.03, *p* = 0.004) were independent predictors of 365-day mortality.

**Conclusions:**

Our study demonstrated that a higher level of CAR was associated with 365-day mortality in AKI patients.

## Introduction

Acute kidney injury (AKI) is a common and serious syndrome in hospitalized patients, referring to an abrupt decrease in glomerular filtration. It is well-know that diagnostic criteria of AKI are based on urine output reduction and serum creatinine rise [1]. The presence of AKI is associated with increased mortality in patients, especially in critical illness [2]. In addition, AKI patients often fail to complete recover renal function and need renal replacement therapy (RRT), which is costly and has a negative influence on patients’ quality of life [3, 4]. Given the high incidence of AKI and its poor outcomes in critical illness, an increasing number of observational studies have been devoted to seeking for reliable predictors of mortality in AKI [5].

The mechanisms of AKI are characterized by inflammation, endothelial dysfunction, hemodynamic alterations and tubular injury. Recently, several studies have suggested that inflammation played an important role in the pathogenesis of AKI [6–8]. Serum C-reactive protein (CRP), an acute-phase protein, markedly increases within hours after inflammation. It could be a useful monitor for inflammatory disease due to the relatively short half-life of approximately 19 hours [9]. Meanwhile, serum albumin has been considered to be a negative acute phase protein in inflammation and associated with AKI development [10, 11]. By merging CRP and albumin into a single index, the CRP to albumin ratio (CAR) is an easily available marker and has been considered to be related to increased risk of AKI in patients after cardiovascular surgery [10]. Moreover, several studies have demonstrated that increased CAR was associated with mortalities in a variety of diseases, including cancer, ischemic stroke, liver failure and infection [12–14]. Therefore, we hypothesized that CAR could predict the outcome of AKI patients. However, it is surprised that little study has examined the relationship between mortality and CAR in AKI patients. In our present study, we aimed to investigate the prognostic value of CAR for mortality in AKI patients.

## Methods

### Study subjects

Medical Information Mart for Intensive Care III (MIMIC III) database (version 1.4) is a large, single-center database comprising information of patients admitted to critical care units at Beth Israel Deaconess Medical Center in Boston between 2001 and 2012 [15]. The establishment of this database was approved by the Institutional Review Boards (IRB) of the Massachusetts Institute of Technology (MIT). This database contains detailed information of distinct patients including demographic characteristics, laboratory data, therapeutic interventions, survival data and more [15, 16]. After completing a training course on the website of National Institutes of Health named ‘Protecting Human Research Participants’, one author was permission to access the database for research purposes (Certification number: 27454094).

**Fig. 1:**
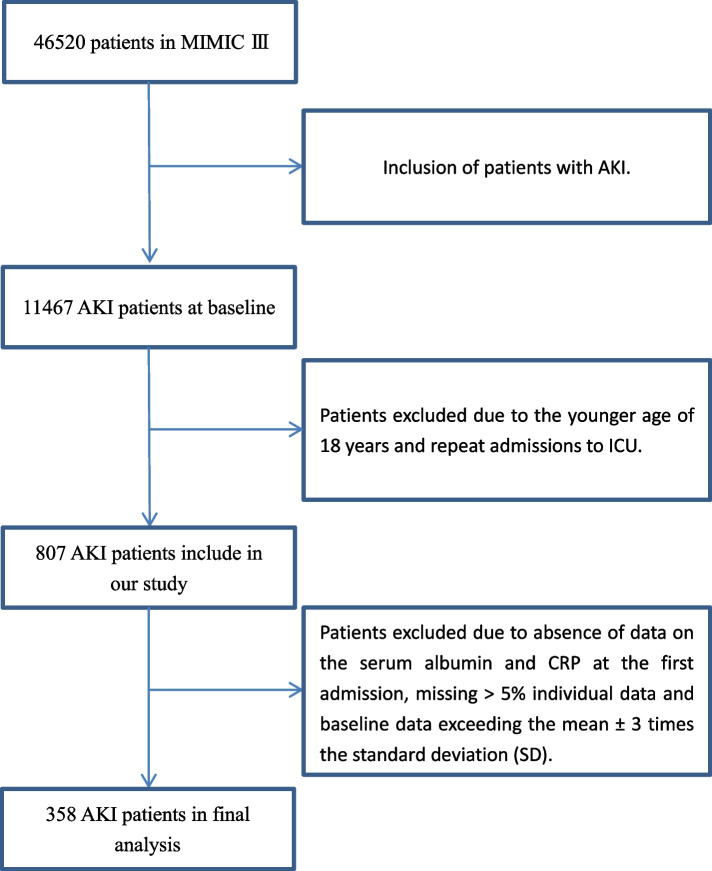
The flowchart of inclusion and exclusion procedure

In this study, we collected 358 consecutive patients with AKI admitted to ICU. Eligible patients met the following inclusion criteria: age 18 years or older at first admission and the primary diagnosis was AKI. The exclusion criteria were: absence of data on the serum albumin and CRP at the first admission, missing > 5% individual data and baseline data exceeding the mean ± 3 times the standard deviation (SD) (Fig. 1).

### Data extraction

All data were extracted from MIMIC III using the Structured Query Language (SQL) with PostgreSQL tools (version 12.0). The data contained clinical parameters, laboratory parameters, comorbidities, and scoring systems. The clinical parameters included age, gender, heart rate, respiratory rate, systolic blood pressure (SBP), diastolic blood pressure (DBP), mean arterial pressure (MAP), percutaneous oxygen saturation (SPO_2_), vasopressin used and RRT. The following laboratory parameters were extracted: CRP, albumin, chloride, anion gap, bicarbonate, lactate, creatinine, potassium, sodium, platelet, glucose and white blood cell (WBC). The comorbidities included the congestive heart failure, hypertension, diabetes, stroke, chronic renal disease, chronic liver disease and malignancy. We also calculated sequential organ failure assessment (SOFA), Glasgow Coma Scale (GCS) and Simplified Acute Physiology Score II (SAPS II). The outcome of our study was 365-day mortality.

### Statistical analysis

Continuous and categorical variables were presented as the mean ± SD and percentage, respectively. One-way ANOVA, X^2^ test, and Mann-Whitney U test were used to compare differences between the clinical characteristics of survivors and nonsurvivors as appropriate. We used One-way ANOVA or Mann-Whitney U test to explore CRP between low and normal albumin group. When results were significant, we added the possible confounders to the Cox regression model to examine the relationship between CAR and outcome. The results were expressed as hazard ratios (HRs) with 95% confidence intervals (CIs). Furthermore, survival curves were calculated using the Kaplan-Meier estimates and comparisons were constructed based on the log-rank test. Receiver operating characteristic (ROC) curves were performed to evaluate the ability of CAR, CPR and albumin to predict mortality in AKI patients. All statistical tests were performed using SPSS 21.0 (SPSS Inc., Chicago, IL). Values of *P* < 0.05 were considered to be statistically significant in all tests.

## Results

### Subject characteristics

A total of 358 eligible participants collected from the MIMIC III database were enrolled into our study. The mean age of participants was 69.43 ± 13.70 years, of which 210 (60.9%) participants were male. The 365-day mortality was 69.6% (*n* = 250); these patients were defined as nonsurvivors. The characteristics of survivors and nonsurvivors are presented in Table 1. Compared with survivors, patients were more likely to be elderly, male and had higher CRP, CAR and SAPS II score, whereas systolic blood pressure was lower in nonsurvival group (all *P*<0.05). There were no significant differences in age and other parameters. Moreover, we divided patients into two groups according to the albumin level. There was no significant difference in CRP between low and normal albumin group.

**Table 1:** Baseline characteristics of study population

Characteristics	Survivors (*n* = 108)	Nonsurvivors (*n* = 250)	*P* value
Clinical parameters			
Age (years)	67.26 ± 14.43	70.37 ± 13.28	0.048
Gender (male) [n(%)]	57 (52.80)	161 (64.40)	0.039
Heart rate (mean ± SD)	87.63 ± 13.64	88.74 ± 18.33	0.571
Respiratory rate (mean ± SD)	19.82 ± 4.87	20.11 ± 4.54	0.588
SBP(mean ± SD)(mmHg)	116.17 ± 21.07	111.83 ± 18.05	0.049
DBP(mean ± SD)(mmHg)	56.28 ± 11.88	57.50 ± 11.57	0.366
MAP(mean ± SD)(mmHg)	75.01 ± 13.62	73.61 ± 12.53	0.348
SPO_2_ (%)	97.03 ± 2.02	96.78 ± 2.94	0.420
Vasopressin used [n(%)]	56 (51.90)	158 (63.20)	0.044
RRT used [n(%)]	6 (5.60)	26 (10.40)	0.140
Laboratory parameters (mean ± SD)			
Lactate (mg/dl)	2.70 ± 1.91	2.91 ± 2.01	0.396
Glucose (md/dl)	156.65 ± 72.61	154.53 ± 70.10	0.796
White blood cell (10^9^/l)	15.58 ± 14.76	14.13 ± 9.89	0.280
Platelet (10^9^/l)	228.41 ± 111.73	229.68 ± 138.27	0.933
Sodium (mmol/l)	138.27 ± 6.13	138.16 ± 6.50	0.882
Potassium (mmol/l)	4.35 ± 0.74	4.35 ± 0.69	0.981
Bicarbonate (mmol/l)	21.95 ± 5.08	22.14 ± 4.98	0.752
Chloride (mmol/l)	105.02 ± 7.89	104.56 ± 7.59	0.603
Anion gap (mmol/l)	15.72 ± 3.55	15.95 ± 3.81	0.586
Creatinine (mg/dl)	2.07 ± 1.45	1.97 ± 1.20	0.504
C-Reactive protein(mg/dl)	72.54 ± 79.13	99.61 ± 83.44	0.004
Albumin (g/dl)	3.07 ± 0.62	2.85 ± 0.58	0.001
CAR	25.05 ± 27.47	36.50 ± 31.96	0.001
Clinical scores (mean ± SD)			
SOFA	5.69 ± 2.83	6.34 ± 3.62	0.097
GCS	13.62 ± 2.47	13.45 ± 2.61	0.561
SAPS II	42.48 ± 11.61	46.37 ± 14.40	0.014
Comorbidity [n(%)]			
Congestive heart failure	40 (37.00)	110 (44.00)	0.220
Hypertension	29 (26.90)	56 (22.40)	0.364
Diabetes	48 (44.40)	98 (39.20)	0.354
Stroke	3 (2.80)	17 (6.80)	0.128
Chronic renal disease	33 (30.60)	75 (30.00)	0.916
Chronic liver disease	11 (10.20)	29 (11.60)	0.697
Malignancy	6 (5.60)	30 (12.00)	0.063

*CAR* C-Reactive protein/albumin, *DBP* diastolic blood pressure, *MAP* mean arterial pressure, *RRT* renal replacement therapy, *SPO2* percutaneous oxygen saturation, *SAPS II* Simplified Acute Physiology Score II, *SBP* systolic blood pressure, *SOFA* Sequential Organ Failure Assessment, *GCS* Glasgow Coma Scale

### Association between CAR and mortality

**Fig. 2:**
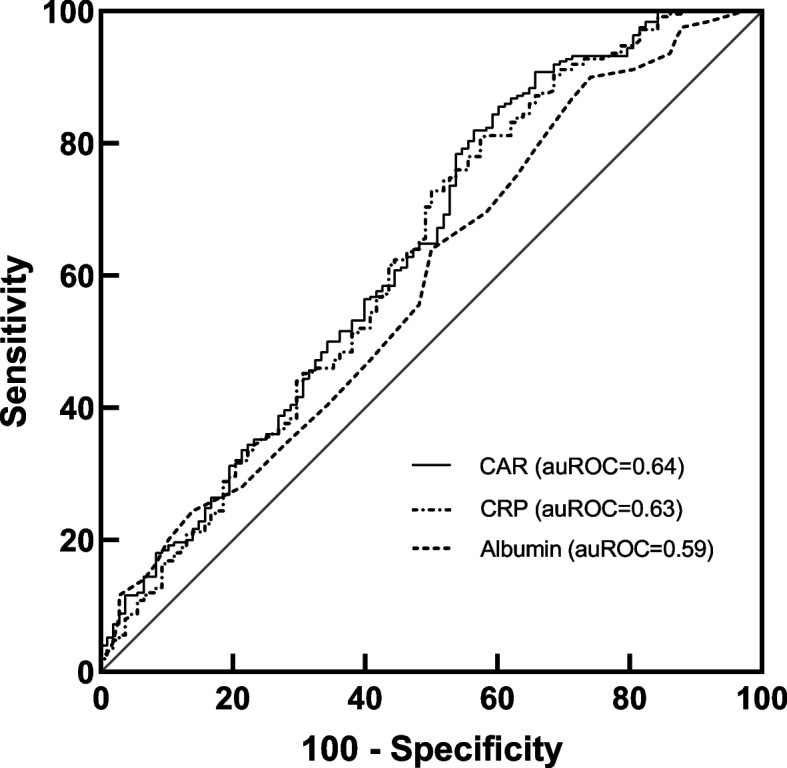
Receiver operating characteristic curves for different models to predict 365-day mortality

Using ROC curves, the optimal CAR cut-off point was 7.23 at admission for predicting 365-day mortality, with high sensitivity and modest specificity [82.0 and 43.5%, respectively; area under the curve (AUC) = 0.64, 95% CI: 0.57-0.70; *P*<0.001]. CAR had a higher prognostic accuracy for 365-day mortality compared to CRP [AUC 0.63 (0.56-0.69), *P*<0.001] and Albumin [AUC 0.59 (0.52-0.66), *p* = 0.007] (Fig. 2).

Using Cox proportional hazard model, we analyzed the influence of age, gender, SBP, Vasopressin used, SAPS II score and CAR on 365-day mortality (Table 2). Patients were divided into two groups according to CAR for survival analysis. The relative risk for mortality was significantly related to CAR (HR =2.04, 95% CI = 1.47-2.85, *P*<0.001 for high CAR) and SAPS II score (HR = 1.02, 95% CI = 1.00-1.03, *P* = 0.004). These results suggested that CAR>7.23 had a strong ability to predict 365-day mortality.

**Table 2:** Cox proportional hazard regression analysis for mortality

Variables	HR	95% CI	*P* value
Age	1.00	1.00-1.01	0.278
Gender			
Female	Reference		
Male	1.40	1.07-1.82	0.014
SBP	1.00	0.99-1.00	0.698
Vasopressin used	1.20	0.90-1.60	0.210
SAPS II	1.02	1.00-1.03	0.004
CAR≤7.23	Reference		
CAR>7.23	2.04	1.47-2.85	<0.001

*CAR* C-Reactive protein/albumin, *SBP* systolic blood pressure, *SAPS II* Simplified Acute Physiology Score II

## Discussion

In our study, we explored the possible association between CAR and mortality in AKI patients. The main finding of our study was that CAR could predict mortality in AKI patients admitted to the ICU. In addition, we found that the higher level of CAR was positively associated with increased risk of 365-day mortality.

The CAR is a combination of CRP and albumin that has been proposed as an inflammation-based prognostic score in diseases and has a potential ability of predicting the prognostic outcome of patients. For example, Park et al. considered that CAR was correlated with high mortality in medical intensive care unit patients [17], which was in line with our results. Another study performed by Ren et al. reported that an increased CAR was closely associated with mortality risk in patients with hepatocellular carcinoma [18]. In addition, Kocaturk et al. suggested that acute ischemic stroke patients with a higher CAR had a lower survival probability [14]. A study conducted by Wang et al. showed that a higher CAR was positively correlated with 30-day mortality in patients with hepatitis B virus-related decompensated cirrhosis [13]. Finally, Llop-Talaveron et al. showed that a high CAR was positively associated with more complications during parenteral nutrition treatment [12]. Taken together, these studies suggested that CAR may be a potentially useful prognostic tool for predicting outcome in patients.

The mechanism of relationship between CAR and mortality in AKI patients may be explained by the inflammation reaction. It has been described that ischemia reperfusion injury and inflammation played important roles in AKI development [6–8, 19]. Ischemic injury to kidney can promote the activation of endothelial renal cells that express adhesion molecules, resulting in inflammatory response [20]. CRP, a marker of inflammation, has been considered to be associated with activated coagulation and platelet system, which may reduce renal blood flow and oxygen delivery to the kidneys [21, 22]. CRP also could mediate the enhanced expression of adhesion molecules, plasminogen activator inhibitor-1 and decreased nitric oxide production [23]. The elevated CRP may cause endothelium dysfunction and alter the vascular equilibrium to vasoconstrictive, proinflammatory and prothrombotic status [24]. Serum albumin is considered a vital protective antioxidant and abundant circulating protein in plasma [25]. The decrease in the serum albumin level may aggravate the renal dysfunction due to oxidative stress damage [26]. Meanwhile, inflammation may reduce albumin concentration by decreasing its synthesis rate. Therefore, the CRP is positively associated with the inflammatory response, and albumin is negatively related to inflammation, resulting in higher CAR. Recently, a cohort study suggested that severe inflammation with increased plasma proinflammatory cytokine could predict mortality in AKI patients [27]. Meanwhile, Doi et al. suggested that the mortality of AKI patients could decrease by using anti-inflammatory cytokines [28]. Taken together, the relationship between CAR and mortality may be associated to the involvement of inflammation.

There are some limitations in our study. First, we only measured CRP and albumin in patients admitted to the ICU once and did not evaluate changes during treatment, which may have a biased influence on the results. Second, the inherent biases of analysis were present in our research because of retrospective study. Third, we did not know the state of nutrition of patients before admitting to the ICU, which may lead to deviation with the actual situation. Fourth, several important data, including insurance status, income and education which may be related to mortality were missing. Fifth, in our study, the dataset between 2001 and 2012 used may have a biased influence on the results. Hence, further studies should be conducted to clarify the relationship between CAR and mortality in AKI patients using newest data. Finally, there was a potential selection bias existing due to our study of patients exclusively collected from a single center, which may limit the generalization of our findings.

## Conclusion

In summary, our study suggested that a higher level of CAR was related to increased risk of 365-day mortality in AKI patients. Our findings need to be confirmed by further prospective studies.

## Data Availability

The datasets used and/or analyzed during the current study are available from the corresponding author on reasonable request.
